# Glutamatergic activation of A1 and A2 noradrenergic neurons in the rat brain stem

**DOI:** 10.3325/cmj.2019.60.352

**Published:** 2019-08

**Authors:** Duygu Gok-Yurtseven, Ilker M. Kafa, Zehra Minbay, Ozhan Eyigor

**Affiliations:** 1 Department of Histology and Embryology, Institute of Health Science, Bursa Uludag University, Bursa, Turkey; 2 Department of Anatomy, Bursa Uludag University Faculty of Medicine, Bursa, Turkey; 3 Department of Histology and Embryology, Bursa Uludag University Faculty of Medicine, Bursa, Turkey; The first two authors contributed equally.

## Abstract

**Aim:**

To analyze the effects of glutamatergic agonists and antagonists on the activation of the A1 and A2 noradrenergic neurons localized in caudal ventrolateral medulla and nucleus tractus solitarii, respectively.

**Methods:**

Rats were injected with glutamatergic agonists – kainic acid, α-amino-3-hydroxy-5-methyl-4-isoxazolepropionic acid (AMPA), or N-methyl-D-aspartic acid (NMDA), and the brain sections were prepared for immunohistochemistry. Before agonist injections, antagonists – 6-cyano-7-nitroquinoxaline-2,3-dione or dizocilpine were administered. The expression of c-Fos, as the neuronal activation marker, and tyrosine hydroxylase (TH), as the marker of noradrenergic neurons was assessed with dual immunohistochemistry. The percentage of c-Fos-positive noradrenergic neurons relative to all TH-positive neurons in the respective areas of the brain stem was calculated.

**Results:**

All three glutamatergic agonists significantly increased the number of the c-Fos-positive noradrenergic neurons in both the A1 and A2 area when compared with control animals. Kainic acid injection activated about 57% of TH-positive neurons in A1 and 40% in A2, AMPA activated 26% in A1 and 38% in A2, and NMDA 77% in A1 and 22% in A2. The injections of appropriate glutamatergic antagonists greatly decreased the number of activated noradrenergic neurons.

**Conclusion:**

Our results suggest that noradrenergic neurons are regulated and/or activated by glutamatergic system and that these neurons express functional glutamate receptors.

Noradrenergic cell groups are neuron groups in the central nervous system (CNS) that contain the neurotransmitter norepinephrine (NA, noradrenalin). The central noradrenergic system (CNA), which consists of noradrenergic cell groups and is located in the brainstem, plays an essential role in the pathogenesis of different neurological disorders and in the regulation of the memory-related, behavioral, and neuroendocrine processes (1-3). In the brain stem, these neurons are localized in the brain areas A1 (caudal ventrolateral medulla, CVLM), A2 (nucleus tractus solitarii, NTS), A4, A5, A6 (locus coeruleus, LC), and A7 (3-5). The noradrenergic neurons in the A1 area are distributed ventrolaterally, while the neurons in the A2 area are distributed dorsomedially. The A1 and A2 neurons express tyrosine hydroxylase (TH), a rate-limiting enzyme in catecholamine synthesis, but are negative for adrenaline-synthesizing enzyme, phenylethanolamine-N-methyltransferase (6,7). A2 noradrenergic neurons are involved in major physiological functions that regulate the cardiovascular and respiratory systems, and gustatory, hepatic, and renal functions (8,9).

Glutamate is the major excitatory neurotransmitter in the mammalian nervous system, which binds and activates ionotropic and/or metabotropic glutamate receptors. The presence of the glutamatergic terminals has been reported in the brainstem (10-13). Glutamate regulates many neuromodulatory processes in the brain stem, including the orchestration of the neuroendocrine, behavioral, and autonomic functions. Reports showing the glutamatergic inputs on noradrenalin neurons and the expression of glutamate receptors by these neurons suggested that glutamate might be involved as a neurotransmitter in the functional regulation of the noradrenergic neurons in the brain stem (13-15).

Noradrenergic neurons in the A1 and A2 areas project to the hypothalamus and regulate distinct neuroendocrine functions (16). A2 group of neurons project to the paraventricular nucleus of the hypothalamus (PVN) and are implicated in stress regulation (17). A1 and A2 neurons control the energy balance (16,18) and food intake (19), and play an important role in the regulation of reproduction through gonadotropin-releasing hormone neurons (GnRH) (20). Since these noradrenergic neurons regulate neuroendocrine functions and express glutamate receptor subunits (21-24), it is important to analyze if these subunits form functional glutamate receptor channels. We hypothesized that, if the glutamate receptors on these neurons are functional, they, as well as the neurons, can be activated by the administration of glutamate agonists. In order to analyze the activation of these centrally located noradrenergic neurons in response to the glutamatergic agonists, N-methyl-D-aspartic acid (NMDA), kainic acid, and α-amino-3-hydroxy-5-methyl-4-isoxazolepropionic acid (AMPA), we employed a double-labeling immunohistochemical approach in which the transient expression of the c-Fos protein was used as a marker of neuronal activation. We also analyzed the blocking effect of glutamate antagonists on neuronal activation in order to show the specificity of agonists’ effects.

## MATERIAL AND METHODS

### Animals and injections

All animal experiments were carried out in accordance with the National Institute of Health Guide for the Care and Use of Laboratory Animals and approved by the Animal Care and Use Committee of the University. Thirty-six 60-day-old female Sprague-Dawley rats weighing 200 to 250 g were used. The rats were maintained at the Bursa Uludag University Experimental Animals Breeding and Research Center and were housed two per cage in a temperature-controlled environment (21°C) with a 12:12-hour light/dark cycle. All the experiments were carried out between 9:00 am and 11:00 am.

The animals were divided into nine groups (n = 4 per group): kainic acid group (intraperitoneal [ip] injections of kainic acid – 2.5 mg/kg in 300 μL distilled water, DW), kainic acid control group (300 μL saline, ip), and kainic acid-6-cyano-7-nitroquinoxaline-2,3-dione (CNQX) group (2 mg/kg CNQX in 300 μL DW, ip, 15 minutes before kainic acid injection), AMPA group (5 mg/kg AMPA in 750 μL DW, ip), AMPA control group (750 μL saline, ip), AMPA-CNQX group (2 mg/kg CNQX in 750 μL DW, ip, 15 minutes before AMPA injection), NMDA group (100 mg/kg NMDA in 2 mL DW), NMDA control group (2 mL saline, ip), and NMDA-dizocilpine (1.5 mg/kg MK-801 in 2 mL DW, ip, 15 minutes before NMDA injection).

### Tissue preparation

Ninety minutes after the injections, the animals were deeply anesthetized and fixed by trans-cardiac perfusion with 4% paraformaldehyde in phosphate buffer, pH 7.4 (300 mL per animal). Brains and brainstems were carefully removed and post-fixed overnight in the same fixative. Thirty-micrometer-thick coronal serial sections throughout the brain stem were cut with a vibratome and collected into Tris-HCl buffer (0.05 M, pH 7.6). The sections were kept in the cryoprotectant solution at -20°C until use.

### Immunohistochemistry

Tris-HCl buffer was used for washing, and blocking buffer (10% normal horse serum, 0.2% triton X-100, and 0.1% sodium azide in Tris-HCl buffer) was used for incubations in order to prevent non-specific binding and to dilute the antibodies. Following 2-h incubation in blocking buffer, sections were transferred into rabbit anti-c-Fos antibody solution at a dilution of 1:20 000 (Chemicon, Billerica, MA, USA) for an overnight incubation. The sections were then exposed to biotin-conjugated donkey anti-rabbit IgG (1:300, Jackson Immunoresearch Laboratories, West Grove, PA, USA) for 2 h, processed with avidin-biotin complex according to the manufacturer’s instructions (ABC Elite Standard Kit, Vector Laboratories, Burlingame, CA, USA) for 45 min, and stained with diaminobenzidine (DAB, 25 mg) and nickel ammonium sulfate (2 g) in the presence of 2 μL hydrogen peroxide in 100 mL Tris-HCl buffer. To terminate the reaction, sections were transferred to Tris buffer again. The c-Fos-immunostained sections were then washed thoroughly in Tris-HCl buffer, incubated in blocking buffer, and exposed to anti-TH antibody (TH, 1:35,000; lot number 41K4829; Sigma-Aldrich, St Louis, MO, USA). Following overnight incubation, sections were exposed to biotinylated secondary antibody, then to avidin-biotin reaction as described above. DAB was used as the chromogensolution for the visualization of the immunochemical complex. Then, sections were collected onto slides, coded, dried, and coverslipped with DPX. Digital photomicrographs of stained cells were taken and analyzed using Olympus BX50 photomicroscope (Olympus, Tokyo, Japan).

### Cell counting

Sections were analyzed at 200 × magnification for cell counting. All TH-positive cells, with and without c-Fos-positive nuclei neurons in the CVLM (A1) and NTS (A2) were counted bilaterally and blindedly in every fourth section between the stereotaxic coordinates of bregma -13.56 mm- bregma -15.96 mm (25).The percentage of c-Fos-positive TH neurons was calculated within each group relative to all TH-positive cells in the same group.

### Statistical analysis

Instead of performing a systematic random sampling, we counted all the labeled cells in the studied areas. The power calculation based on an effect size of 0.8, a standard deviation of 12, and an alpha level set at 0.05 showed that the required sample size to obtain a power of 0.8 was 4 animals per group (36 animals for 9 groups).

Shapiro Wilks test was used for normality testing. The percentage of the c-Fos positivity in TH-positive cells is expressed as mean ± standard deviation. The significance of differences between the groups was assessed with Kruskal-Wallis test. The level of statistical significance was set at *P* < 0.05. Statistical data analysis was performed by IBM SPSS, 23.0 (IBM Corp. Armonk, NY, USA).

## RESULTS

Injections of agonists or antagonists did not cause adverse reactions or neurological problems, and there was no mortality during the experiments. Drug-induced spontaneous neurological effects (eg, shivering) were negligible, and no pathological changes were observed in rats' brains, brain stems, and spinal cords after the removal.

**Figure 1:**
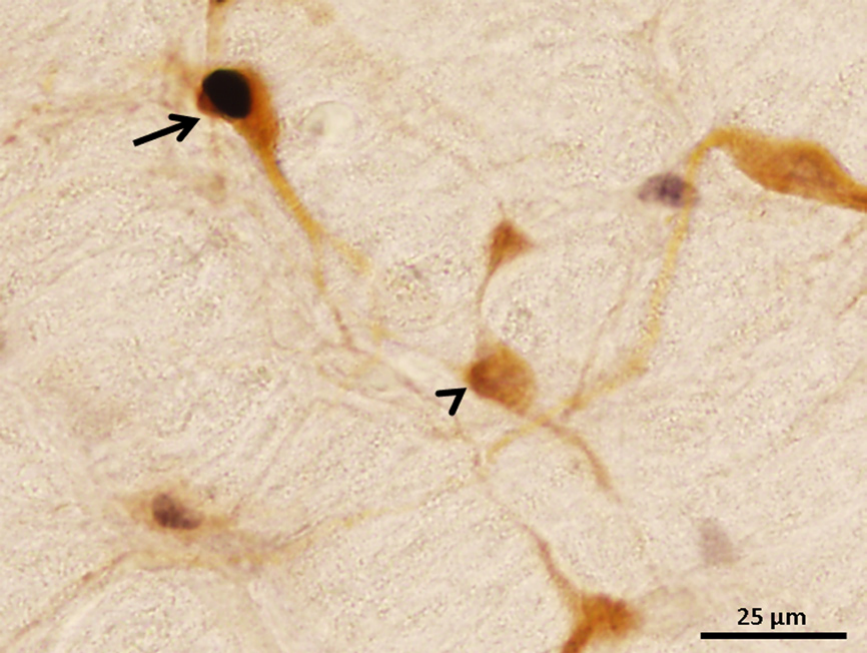
Representative c-Fos-positive and c-Fos-negative noradrenergic neurons. The arrow indicates c-Fos positivity (dark-blue) in the nucleus of a tyrosine hydroxylase (TH)-positive (brown) neuron. The arrow-head indicates a representative c-Fos-negative noradrenergic neuron.

**Figure 2:**
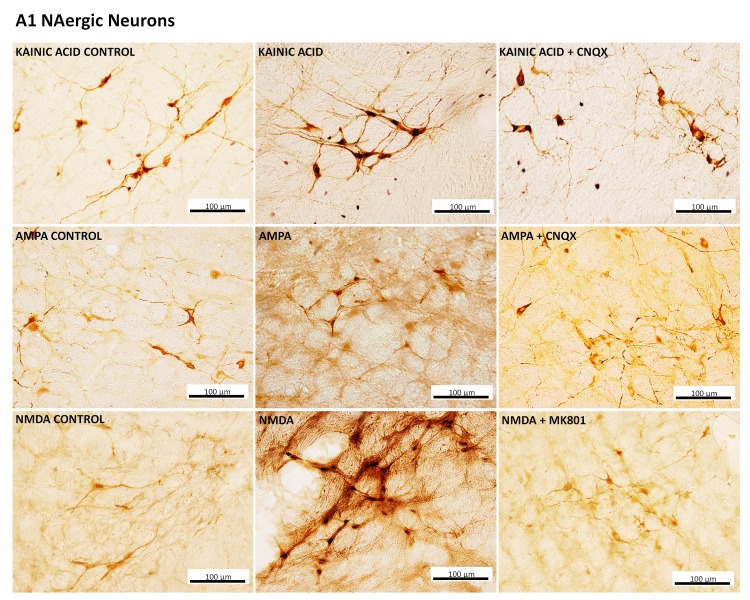
Representative images of A1 noradrenergic neurons of the brainstem double-stained for c-Fos and tyrosine hydroxylase (TH). The effect of kainic acid (upper set), α -amino-3-hydroxy-5-methyl-4-isoxazolepropionic acid (AMPA) (middle set), and N-methyl-D-aspartic acid (NMDA) (lower set) is shown. Control groups are shown on the left, the agonist injected groups in the middle, and antagonist-injected groups on the right.

**Figure 3:**
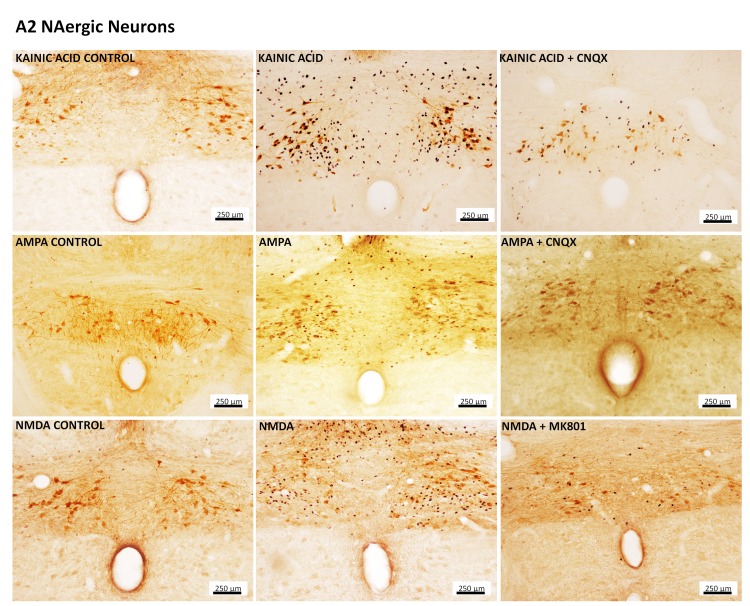
Representative images of A2 noradrenergic neurons of the brainstem double stained for c-Fos and tyrosine hydroxylase (TH). The effect of kainic acid (upper set), α -amino-3-hydroxy-5-methyl-4-isoxazolepropionic acid (AMPA) (middle set), and N-methyl-D-aspartic acid (NMDA) (lower set) is shown. Control groups are shown on the left, the agonist injected groups in the middle, and antagonist-injected groups on the right.

**Figure 4:**
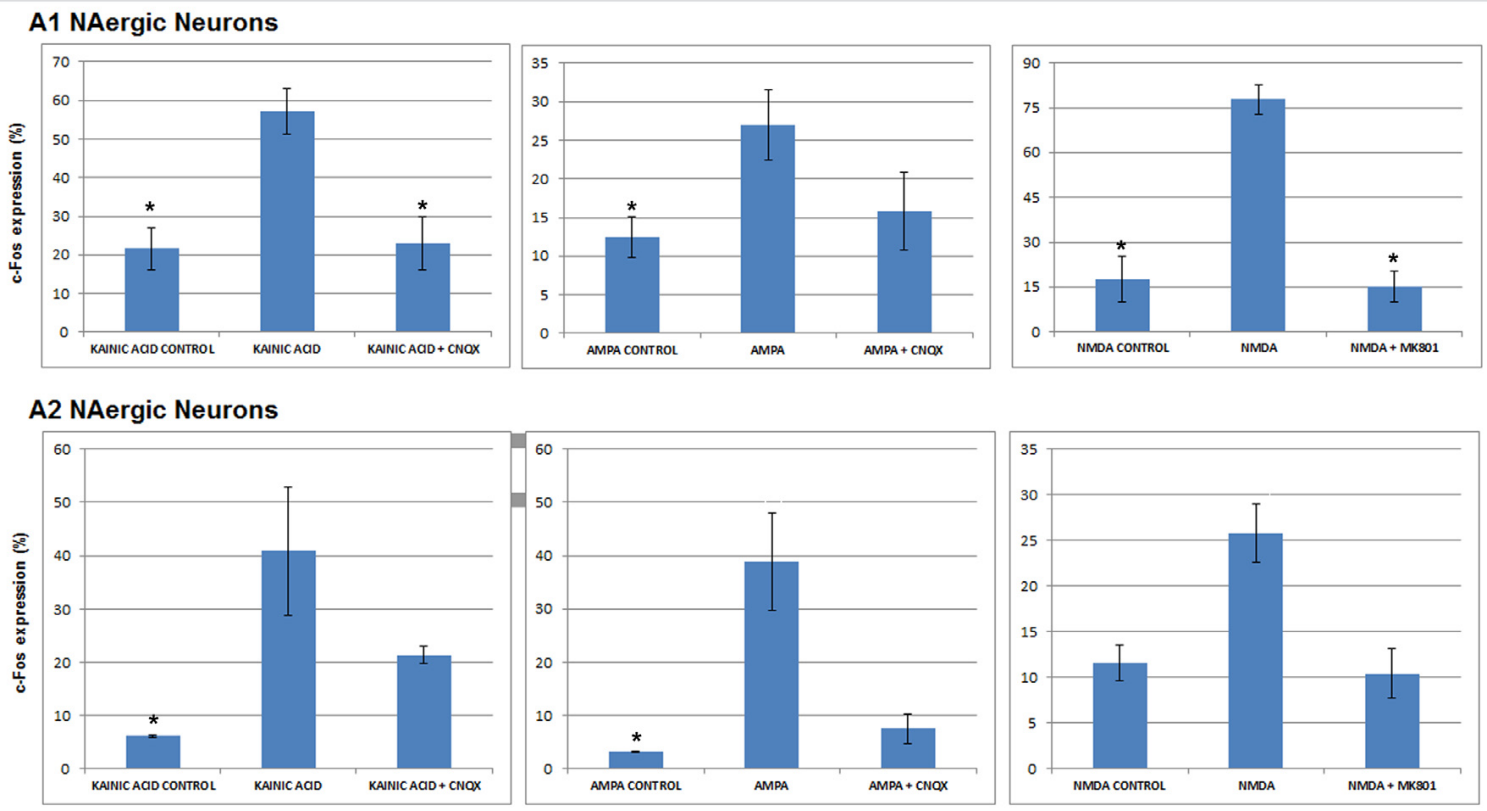
The percentages of c-Fos-positive tyrosine hydroxylase (TH) neurons relative to all TH-positive cells. The figure summarizes the effects of agonists and antagonists when compared with controls. Agonist injections activated significantly more neurons when compared with the control animals. Antagonist injections blocked this activation and the percentage of activated neurons decreased significantly. Asterisk indicates the statistical significance when compared with agonist-injected groups. See Table 1 for actual *P* values.

TH positivity was visualized by brown reaction product with chromogen in the cytoplasm and c-Fos positivity by dark-brown/black (nickel intensified chromogen) in the nuclei (Figure 1). Double immunohistochemistry for c-Fos and TH showed that the glutamatergic system strongly influenced these two separate but neurochemically related neuron groups. All three glutamatergic agonists (kainic acid, AMPA, and NMDA) increased the number of c-Fos+/TH^+^ cells in both CVLM and NTS compared with control groups (*P* < 0.05). These effects were also blocked by antagonists that are specific for each agonist (CNQX for kainic acid and AMPA, and MK-801 for NMDA). In A1 neurons, NMDA caused the greatest increase in the percentage of c-Fos expressing TH-positive neurons (77.93 ± 7.78%). This increase was significantly greater when compared with the control group (17.6 ± 2.78%, *P* = 0.014). Kainic acid activated about 57% of TH-positive neurons (57.11 ± 4.90, significantly more than in the control group: 20.45 ± 10.47%, *P* = 0.011), whereas AMPA activated about 26% of neurons (26.96 ± 5.55%, significantly more than in the control group: 12.44 ± 5.20%, *P* = 0.012). In A2 group, both kainic acid (40.9 ± 19.49% vs 6.17 ± 3.92%, *P* = 0.011) and AMPA (38.89 ± 3.47% vs 3.21 ± 0.03%, *P* = 0.003) significantly increased the number of activated neurons compared with the control group, but in the NMDA group the increase was not significant (*P* = 0.087). The injections of specific antagonists prior to agonists clearly decreased the percentage of double-labeled neurons in all groups. However, significant difference was only detected in the A1 group when kainic acid+CNQX and NMDA+MK-801 were used (Table 1, Figure 2, Figure 3, and Figure 4)

**Table 1:** The percentages of c-Fos-positive tyrosine hydroxylase (TH) neurons relative to all TH-positive cells in all groups (mean ± standard deviation). *P* values pertain to the comparison with the kainic acid, α-amino-3-hydroxy-5-methyl-4-isoxazolepropionic acid (AMPA), or N-methyl-D-aspartic acid (NMDA)-injected groups*

	c-Fos expression (%)			
	A1 noradrenergic neurons	*P*	A2 noradrenergic neurons	*P*
Kainic acid control	20.45 ± 10.47	0.011	6.17 ± 3.92	0.011
Kainic acid	57.11 ± 4.90		40.9 ± 19.49	
Kainic acid + CNQX	22.94 ± 0.58	0.031	21.36 ± 14.14	0.202
AMPA control	12.44 ± 5.28	0.012	3.21 ± 0.03	0.003
AMPA	26.96 ± 5.55		38.89 ± 3.47	
AMPA + CNQX	15.85 ± 6.59	0.077	7.6 ± 0.74	0.239
NMDA control	17.6 ± 2.78	0.014	11.59 ± 6.15	0.087
NMDA	77.93 ± 7.78		25.83 ± 6.57	
NMDA + MK801	15.12 ± 1.41	0.024	10.4 ± 6.08	

*CNQX – cyano-7-nitroquinoxaline-2,3-dione; MK801 – dizocilpine.

## DISCUSSION

The present study showed that the noradrenergic neurons in the A1 and A2 areas of the brain stem were activated by glutamatergic agonists. The results also demonstrated that glutamatergic antagonists can specifically block this activation, suggesting that functional glutamate receptors are expressed in the noradrenergic neurons of the CVLM and NTS. Considering the extensive networks and widespread connections of the noradrenergic system, it can be deduced that its altered activity may lead to extensive changes in other brain parts, including the basal brain, forebrain, and limbic system. The present study suggests that noradrenergic neurons are glutamate-receptive, which is also supported by other reports (21-23). In the NTS, both NMDA and AMPA receptors are expressed by A2 group of neurons (26,27), while in the CVLM, AMPA receptor subunits are synthesized by the A1 group of neurons, which they project to the hypothalamus (21).

The central noradrenergic system is connected with hypothalamic pituitary adrenal axis mainly through PVN and its corticotropin-releasing hormone neurons (28). Hypothalamic PVN and supraoptic nuclei (SON) have projections from A1 and A2 neurons of the brainstem, and magnocellular neurons of these two essential hypothalamic nuclei are likely the principal targets of the caudal noradrenergic system (29). These projections, which are arising from the medullary noradrenergic neurons to the hypothalamus, may regulate neuroendocrine functions of the diencephalon, and, as our results revealed, may be under the control of glutamatergic neurons. Also it is shown that NA injections into the PVN stimulate food intake and that the NA levels are interrelated with leptin levels, which clearly indicate the relationships among noradrenaline, appetite, and energy state (30). Glutamatergic system can contribute to these effects as an indirect regulator through the central noradrenergic neurons (31,32).

It is well known that neurons in many hypothalamic nuclei, including SON and PVN, receive substantial inputs from noradrenergic neurons of the brainstem (28). Excitatory type connections of the A2 neurons of the NTS to oxytocin neurons and of the A1 neurons of the CVLM to vasopressin neurons have been documented previously (33). In addition to these direct projections, A1 neurons are indirectly connected to oxytocin neurons via NTS, and a major role in these connections is played by α1 noradrenergic receptors (34). Previous studies demonstrated that the electrophysiological activation of the A1 neurons excitated vasopressin secreting neurons of the hypothalamus and increased blood pressure (35,36). It is plausible to suggest that the signals that arrived to oxytocin and vasopressin neurons are subject to glutamatergic regulation at the level of the brain stem, since the present study showed that glutamate can activate A1 and A2 noradrenergic neurons.

The endogenous glutamatergic signals may arrive to the brain stem noradrenergic neurons from the periphery. The literature shows that the glutamatergic neuronal endings make synaptic formations on NTS A2 neurons (37,38). It is also reported that the stimulation of the uterine cervix is translated into two daily prolactin surges, through the activation of PVN-projecting neurons in the A1, A2, and LC (39). After the cervical stimulation, an increase in the percentage of TH/FG+ double-labeled neurons expressing c-Fos was shown in the A1 and LC. These data provide evidence of a functional pathway of A1 and LC neurons projecting to the PVN that conveys an excitatory signal from the periphery, which may be glutamatergic, as the present study showed that these neurons can be activated by glutamate.

In addition, some noradrenergic terminals on the GnRH cell bodies also originate ipsilaterally from the caudal portion of the medullary noradrenergic system and may be related to GnRH release and sexual behaviors. These connections appear to originate from both A1 and A2 groups (40). The present study suggests that some actions of the GnRH system may be indirectly regulated by glutamatergic system through the connections of central noradrenergic system neurons.

Our results showed that the glutamate receptors expressed by noradrenergic neurons in the A1 and A2 areas were functional. The activation of these neurons by glutamatergic challenge was assessed by transient c-Fos expression, which was used as a neuronal activation marker. Our results showed that glutamate agonists can bind and activate these receptors, which in turn activates the neurons and possibly results in noradrenaline synthesis and/or secretion (which is not assessed in the present study). This activation suggests that noradrenergic neurons possess functional glutamate receptors. The effects of the antagonists in terms of decreased number of activated neurons after agonist challenge suggest that the activation is specific to glutamate receptors. Since the antagonists block the glutamate receptors on noradrenergic neurons, it is plausible that the agonists cannot bind to their receptors in order to activate the neurons. Although all chemicals used in this study pass the blood-brain barrier, the peripheral injection presents a limitation to the study in terms of assessing the agonists’ and antagonists’ effects when administered centrally.

Although molecular and synaptic features of the central noradrenergic system are not sufficiently investigated, and although this system is scattered in the brainstem, it is very effectively and significantly linked to important CNS structures and regions, including the hypothalamus. Our results showed that three different glutamate agonists in the brainstem of adult rats activate two separate noradrenergic neuronal groups that are known to regulate discrete hypothalamic nuclei. Here, we suggest that some of the noradrenergic effects on the hypothalamic neurons are regulated by glutamate at the brain stem level. Further studies are required to unravel the chemical relations between the glutamatergic and noradrenergic systems in respect to their locations, distinct projections to the autonomic and limbic structures, as well as their collaborated molecular, hormonal, and behavioral functions.
